# Osteoporosis and adult spinal deformity—a review for spinal surgeons by the SRS adult spinal deformity task force on senescence

**DOI:** 10.1007/s43390-026-01342-w

**Published:** 2026-04-28

**Authors:** Elizabeth Lord, Matthew Vincent, Corey T. Walker, Joseph Osorio, Camilo Molina, Kristen Jones, Miranda van Hooff, Alekos Theologis, Mitsuru Yagi, Laurel Blakemore, Suken Shah, Serena Hu, Marinus de, Kleuver, Javier Pizones, Michael Kelly, Ferran Pellise, Christopher Ames, Robert Eastlack

**Affiliations:** 1 Department of Orthopedics, University of California Los Angeles, Los Angeles, CA US; 2 Department of Neurosurgery, Cedars-Sinai Medical Center, Los Angeles, CA US; 3 Department of Neurosurgery, University of California San Diego, San Diego, CA US; 4 Department of Neurosurgery, Washington University in St. Louis, St. Louis, MO US; 5 Department of Neurosurgery, Duke University, Durham, NC US; 6 Department for Scientific Research, Sint Maartenskliniek, Ubbergen, Nijmegen, Netherlands; 7 Department of Orthopedic Surgery, University of California, San Francisco, CA US; 8 Department of Orthopedic Surgery, International University of Health and Welfare, Tochigi, Japan; 9 Department of Orthopedic Surgery, Pediatric Specialists of Virginia, Fairfax, VA US; 10 Department of Orthopedic Surgery, Nemours Children’s Hospital, Wilmington, DE US; 11 Department of Orthopedic Surgery, Stanford University, Redwood City, CA US; 12 Department of Orthopedic Surgery, Radboud University, Nijmegen, Netherlands; 13 Department of Orthopedic Surgery, La Paz University Hospital, Madrid, Spain; 14 Department of Orthopedic Surgery, Rady Children’s Hospital, San Diego, CA US; 15 Department of Orthopedic Surgery, Barcelona Spine Institute, Barcelona, Spain; 16 Department of Neurosurgery, University of California San Francisco, San Francisco, CA US; 17 Department of Orthopedic Surgery, Scripps Clinics, La Jolla, CA US; 18 Department of Orthopaedic Surgery, University of California Los Angeles, 1225 15th St, Santa Monica, CA 90404 US

**Keywords:** Osteoporosis, Osteopenia, Adult spinal deformity, Bisphosphonates, Parathyroid hormone receptor agonists

## Abstract

**Purpose:**

To explore the understanding of osteoporosis in the context of adult spinal deformity (ASD) surgery with a focus on diagnosis, surgical risks, non-operative, and operative treatment techniques.

**Methods:**

A comprehensive literature search was performed. Articles were identified using the search terms “osteoporosis,” “adult spinal deformity,” “scoliosis,” and “spine surgery.” Relevant studies were selected based on their focus on the intersection of these topics with an emphasis on utility in spine surgery.

**Results:**

This review discusses osteoporosis diagnosis, surgical risks, and non-operative, and surgical treatments in the context of ASD surgery with a focus on clinical applications. Areas for future growth are highlighted.

**Conclusion:**

As adult spinal deformity (ASD) patients increase globally, the Scoliosis Research Society (SRS) formed a task force to improve understanding of osteoporosis and its impact on this vulnerable population. This review brings together the latest research on the topic and highlights this area as one of critical importance and growth in the field.

## Introduction

Adult spinal deformities (ASD) are one of the fastest growing and most common medical problems in the elderly. The prevalence is estimated at 27.5 million people in the United States with a significant economic burden [1]. The costs of non-operative management are substantial, often without any improvement in health status; and the costs of operative management reach over $100,000 per primary surgery and $55,000 for revision [2–4]. There are robust data indicating improved patient quality of life for patients who undergo surgery, and accordingly, adult spinal deformity surgery prevalence is increasing significantly [5–9]. The Scoliosis Research Society (SRS) assembled a Task Force to improve understanding of senescence, frailty, and aging in the population of adult spinal deformity patients. This purpose of this paper is to explore current knowledge of osteoporosis and bone density; understand the assessment and treatment of poor bone density in this population; and review the relationship between poor bone density with patient outcomes and complications.

## Search Strategy

A systematic literature search was performed using PubMed and MEDLINE databases. The following search terms were queried: “osteoporosis,” “osteopenia,” “adult spinal deformity,” “scoliosis,” “spine surgery,” “spinal fusion,” “bone mineral density,” “bisphosphonates,” “teriparatide,” “romosozumab,” “abaloparatide,” “denosumab,” “calcitonin,” “raloxifene,” “vitamin D,” “mechanical failure,” “proximal junctional kyphosis,” and “bone health optimization.” Searches were limited to publications in English through December 2024. Titles and abstracts were screened for relevance and complete review performed for all potentially eligible articles. Studies were selected based on applicability to the relationship of osteoporosis and ASD surgery, with priority given to randomized controlled trials, prospective cohort studies, and systematic reviews. Retrospective studies, case series, and animal studies were included where higher-level evidence was unavailable.

### Osteoporosis

Osteoporosis is a decrease in bone mineral density (BMD), microarchitectural disruption that leads to decreased bone strength and increased fracture risk. The clinical diagnosis of osteoporosis is made by a fragility fracture of the spine, hip, distal radius; or a T-score of ≤  − 2.5 standard deviations based on dual-energy x-ray absorptiometry (DXA). As many patients have full spine imaging but no DXA, it is critical that spine surgeons assess vertebral bodies for remote compression fractures and, thereby, a diagnosis of osteoporosis.

Osteoporosis can occur as a primary process or as a secondary outcome of underlying pathologic and pharmacologic etiologies. Primary osteoporosis disrupts the equilibrium of anabolic and catabolic bone metabolism. Bone resorption overtakes remodeling, and overall BMD and skeletal mass decrease. [10, 11] Individual risk factors for primary osteoporosis include advanced age, female sex, early menopause, lower body mass, white or Asian race, family history, sedentary lifestyle, and tobacco use.

Although primary osteoporosis is the most common cause of osteoporosis in post-menopausal women, up to 30% of post-menopausal osteoporosis is secondary osteoporosis, [12] and a workup should be performed in all low BMD patients to investigate treatable underlying etiologies. The most common etiologies of secondary osteoporosis disrupt the same mechanisms of bone formation responsible for primary osteoporosis; these can directly affect imbalances in Vitamin D and calcium metabolism, as well as changes in proliferation and differentiation of osteoblasts, osteoclasts, and osteocytes.

Glucocorticoid-induced osteoporosis is the most common cause of secondary osteoporosis. It can be secondary to increased endogenous production as in Cushing’s disease but is more commonly due to iatrogenic exogenous glucocorticoid administration [13]. Glucocorticoids increase resorption and decrease bone formation through multiple pathways, as they induce osteoclast differentiation, deplete pre-osteoblast cell reserves, inhibit mature osteoblast function, and inhibit gonadotropin release thereby lowering sex hormone production. Other pathologic causes of secondary osteoporosis include hyperthyroidism, hyperparathyroidism, hypogonadism, gastrointestinal malabsorption, autoimmune diseases, anorexia, and disruptions in renal calcium excretion. In the case of autoimmune diseases, patients often receive glucocorticoids as treatment and thus have multifactorial low BMD.

### Dual-energy X-ray absorptiometry

In use since 1987, DXA remains the gold standard modality for evaluating BMD, and thus diagnosing and tracking osteoporosis. For female patients and all adults over 50, DXA values are referenced to young, healthy female controls, producing a statistical T-score equivalent to the number of standard deviations from the control mean. The World Health Organization diagnostic criteria define osteopenia as a T-score between − 2.5 and − 1.0 at any site, while osteoporosis is a T-score below − 2.5. The presence of a fragility fracture qualifies as severe osteoporosis. Males under 50 may be referenced to age- and sex-matched controls producing a Z-score [14]. These definitions are used in concert with the FRAX fracture risk assessment algorithm to guide treatment initiation [15].

Degenerative and adult spinal deformity patients can have artificially inflated DXA-based lumbar spine BMD measurements despite low true BMD. In degenerative spine patients, sclerosis and osteophyte formation skew results higher; in ASD patients, the increased load bearing along curve concavities induces remolding per Wolff’s law, increasing measured BMD [16]. This can obscure low BMD in the cancellous bone of the vertebral body and pedicle, [17] a potential limitation to the use of DXA-based BMD measurements in spine surgery particularly in the setting of deformity. Prior instrumentation or larger body habitus can also distort DXA measurements of the lumbar spine [7]. These may be mitigated by instead analyzing a greater number of peripheral sites on DXA, including total hip and distal radius scores.

### Hounsfield units

Advanced imaging studies that are commonly performed as part of an adult spinal deformity workup represent opportunities to augment BMD evaluation given some limitations of DXA and the inherent cost and radiation of an additional study. Computed tomography (CT) scans allow for radiodensity evaluation based on Hounsfield units (HU), a relative measurement of radiodensity with a reference point of 0 for distilled water and − 1000 for air. HU’s may be computed post hoc without changes to imaging protocol, though different acquisition voltage changes values [18]. While this analysis was introduced as a method of opportunistic screening of all comers who underwent CT chest/abdomen, recent focus on spine patients and specific operative concerns has yielded promising results as another tool for preoperative evaluation. There are not yet generally accepted osteoporotic HU cutoff values or direct correlation with gold-standard DXA T-scores [19], and clinical validation is ongoing. In ASD surgery early data indicates HU of 120 – 163 in the upper instrumented vertebra (UIV) to minimize risk of proximal junctional kyphosis (PJK) [20, 21]. There is growing evidence that HU-based evaluation may identify more cases of decreased BMD where DXA T-scores underestimate density loss, and is an important area for future study [10, 22, 23].

### Vertebral bone quality

Vertebral bone quality (VBQ) is a similar metric derived from magnetic resonance imaging (MRI) studies where the median T1 signal intensity of L1-L4 vertebral bodies is divided by the T1 signal intensity of cerebrospinal fluid at L3. VBQ’s do not depend on scanner setup and show moderate correlation to femoral neck and overall lowest T-scores on DXA scan [24]. Similarly to CT HU, there have been several proposed VBQ cutoff values for osteopenia and osteoporosis [12]. In at least one study, patient factors including hyperlipidemia have resulted in scores suggestive of lower BMD than that measured by DXA [25]. Whether this is a deficiency of the measurement or a true finding of hyperlipidemia’s effect on bone architecture requires further study.

### Trabecular bone score

Trabecular bone score (TBS) is an emerging clinical tool that provides an indirect measure of the microarchitectural quality of bone, supplementing standard BMD assessments obtained from DXA. Unlike BMD—which quantifies the mineral content—TBS uses the pixel-level variations in the DXA image to assess trabecular connectivity and texture, giving clinicians a deeper insight into fracture risk [26]. TBS complements BMD by refining fracture risk stratification when BMD alone may not fully capture impaired bone quality (e.g., in older adults or individuals with specific conditions such as diabetes) [27].

While TBS is not a standalone diagnostic criterion for osteoporosis, it has been stratified into ranges for clinical interpretation:

o TBS ≥ 1.350: Indicates relatively preserved trabecular microarchitecture.
o TBS 1.200–1.350: Suggests partially degraded trabecular structure.
o TBS < 1.200: Reflects more severely compromised or “degraded” microarchitecture [28].

TBS can be used alongside clinical risk factors, BMD T-scores, and tools like FRAX to improve the predictive accuracy for osteoporotic fractures, especially in postmenopausal women and the elderly for risk assessment [29]. It is also useful for monitoring therapy: TBS may help track the efficacy of osteoporosis treatments, providing additional feedback on improvements or maintenance of trabecular bone health.

### Risks of ASD surgery with osteoporosis

Surgical treatment of deformity requires fusion, which is an extensive process of bone resorption, formation, and remodeling. This is a metabolically onerous process that is heavily reliant of bone quality. Adult spinal deformity and osteoporosis often coexist, with progressive deformities related to the instability of osteoporotic bone itself [30, 31]. Noh et al. demonstrated that consideration of BMD improves the prediction of mechanical complications of the global alignment and proportion (GAP) score [32]. Multiple studies have demonstrated increases in complications for ASD patients with osteoporosis who undergo surgery, including increase in any complication, reoperation, and proximal junctional failure (PJF) [33]. Reasons for this are multifactorial. Osteoporotic patients fuse at lower rates than patients with normal bone density [34, 35]. Screw purchase is known to be weaker in osteoporotic bone compared to normal bone [36–38]. Poor bone quality is also correlated with increased risk of instrumentation failure [39]. Gupta et al. showed that the prevalence of revision surgeries at 24 months for ASD patients with osteoporosis was significantly higher than those without; 40.5% versus 28% [40]. These findings highlight the need for continued optimization of osteoporotic ASD patients before, during, and while healing from surgery.

### Perioperative medical optimization

Medical treatment can reduce bone loss and even improve bone density. Thus, preoperative medical treatment to optimize bone density prior to surgery has been suggested. The American Orthopaedic Association’s Own the Bone campaign highlighted the importance of secondary fracture prevention program in the setting of osteoporotic fractures and helped define bone health optimization (BHO) [41]. BHO is the practice of assessing bone quality, identifying and correcting modifiable issues, and treating poor bone quality [41]. This is in response to concern that, while awareness of osteoporosis and poor bone density are increasing, appropriate treatment by surgeons is not. Kuprys et al. performed a retrospective review of patients undergoing spinal deformity surgery and found an increase in documentation of bone health discussions, but no corresponding increase in bone quality interventions [42].

Anderson et al. discussed their experience with BHO in a retrospective review 104 patients who underwent optimization prior to lumbar spine surgery [43]. These patients were referred to the program based on presence of risk factors for osteoporosis including previous fractures, known osteoporosis, or bone density measurement on opportunistic CT. The mean hip T-score on DXA was -2.09 with 32.1% of patients having osteoporosis, 59.2% osteopenia, and 8.7% normal bone density. Anabolic therapy was prescribed for 42% of patients and antiresorptive therapy for 30%.

There are several classes of medications to consider, most research of their efficacy focuses on fracture risk reduction, with all treatments having at least one placebo-controlled trial [44]. Notably, much of the data discussed here does not specifically address ASD patients or surgery, but rather bone density or the less challenging fusion environment of single level lumbar spine fusion. Still this research can be cautiously extrapolated to apply in some ASD scenarios Table 1.

**Table 1:** Comparison of diagnostic modalities for bone mineral density assessment in adult spinal deformity

Modality	Advantages	Key thresholds / findings	Limitations in ASD	Clinical recommendations
DXA (dual-energy X-ray absorptiometry)	Gold standard; T-score & Z-score	T-score ≤ − 2.5 = osteoporosis; − 2.5 to − 1.0 = osteopenia	Inflated values in ASD pts; distorted by prior instrumentation or large body habitus	All women ≥ 65; men ≥ 70; younger pts with risk factors. Use hip & distal radius in ASD pts to reduce artifact
Hounsfield units (CT-based)	Opportunistic; uses existing CT scans; no added cost/radiation	HU 120–163 at UIV associated with lower PJK risk; ongoing validation	No universally accepted osteoporotic cutoff	Any ASD pt with available CT. Valuable adjunct when DXA may overestimate density
Vertebral bone quality (VBQ – MRI)	No radiation; uses existing MRI; no additional scanning needed	Moderate correlation with DXA femoral neck T-scores	Comorbidities (e.g., hyperlipidemia) may skew results; further validation needed	ASD pts with MRI obtained during workup. Useful when CT/DXA unavailable or unreliable
Trabecular bone score (TBS)	Derived from DXA; adds microarchitecture data; no extra scan	TBS ≥ 1.350 normal; 1.200–1.349 partial degradation; < 1.200 severe degradation	Not standalone diagnostic; not universally available on all DXA platforms	Supplement DXA, especially in postmenopausal women, elderly, diabetics; useful for treatment monitoring

*DXA* dual-energy X-ray absorptiometry *ASD* adult spinal deformity, *HU* hounsfield units, *UIV* upper instrumented vertebra, *PJK* proximal junctional kyphosis, *VBQ* vertebral bone quality, *TBS* trabecular bone score

### Vitamin D

Vitamin D is required for dietary calcium absorption, mineralization of bone matrix, and collagen cross linking. It is commonly deficient in spine surgery patients [45]. It is recommended for osteoporotic patients to limit bone resorption in the setting of low serum calcium.

Xu et al. compared TLIF patients who underwent postoperative vitamin D supplementation to those who did not and found significantly great fusion rates in the supplemented group (95% vs 65%) in addition to greater ODI [46]. Ravindra et al. performed an observational study of patients undergoing spinal fusion in the cervical, thoracic, and lumbar spines, adjusting for demographics and bone graft materials. They found that vitamin D deficiency was a predictor of prolonged time to fusion and pseudarthrosis [47].

Vitamin D is likely a useful adjuvant for other bone density medications, but further research is required Table 2.

**Table 2:** Summary of pharmaceutical treatment options for bone health optimization in adult spinal deformity surgery

Agent	Class	Mechanism	Route	Timing in ASD	Key spine surgery evidence	Key side effects/cautions	Clinical recommendation
Vitamin D	Adjuvant/supplement	Antiresorptive support	Oral	Deficiency correction; routine supplementation	Higher fusion rates (95% vs 65% in TLIF); reduces pseudarthrosis risk	Few; safe in renal pts at standard doses	All ASD pts; baseline 25-OH Vit D level; supplement to ≥ 30 ng/mL
Calcitonin	Antiresorptive	Inhibits osteoclasts; decreases serum calcium	Nasal or SC	Postmenopausal osteoporosis (secondary use)	Mixed results in animal fusion studies; limited clinical spine fusion data	Nausea; rhinitis (nasal); tachyphylaxis with long-term use	Limited role in ASD perioperative optimization; not a primary recommendation
Bisphosphonates (Alendronate, Zoledronic Acid)	Antiresorptive	Inhibit osteoclasts; remain in bone for decades	Oral (alendronate) / IV (zoledronic acid)	Continue perioperatively; do not withhold for surgery	Do not inhibit fusion; reduce VCF, cage subsidence, screw loosening (ZA RCT data)	Atypical femoral fracture (rare); ONJ (rare); GI upset (oral)	Continue in all pts already on BP; initiate if anabolic therapy unavailable or post-anabolic sequencing
SERMs (Raloxifene)	Antiresorptive	Activate estrogen receptors in bone; inhibit osteoclasts	Oral	Postmenopausal women with osteoporosis	Reduces vertebral fractures; preserves BMD; limited spine fusion data	DVT risk; hot flashes; not for premenopausal women	Consider in postmenopausal women intolerant of bisphosphonates; clinical spine fusion data lacking
Denosumab	Antiresorptive	Monoclonal Ab to RANKL; inhibits osteoclastogenesis	SC injection every 6 months	Postmenopausal osteoporosis; ADT/AI-associated bone loss	Increased pedicle screw pullout strength at 12–24 months; combination with teriparatide may accelerate fusion	Rebound bone loss if discontinued without sequencing; hypocalcemia; ONJ	Ensure follow-on antiresorptive therapy after discontinuation; promising in combination protocols
Teriparatide [rhPTH(1–34)]	Anabolic	Stimulates osteoblasts > osteoclasts; increases BMD, bone mass & strength	SC injection daily (up to 24 months)	Recommended ≥ 3 months preop and continued 1 year postop for osteoporotic ASD pts	Reduced PJK (4.6% vs 15.2%); faster fusion; fewer reoperations; outcomes equivalent to normal BMD pts in deformity surgery	Black box warning (historical osteosarcoma risk in rats; rescinded in US); cost; limited to 24 months lifetime use	First-line anabolic for osteoporotic ASD pts; strongest evidence base for deformity surgery
Abaloparatide	Anabolic	PTH-related protein analog; anabolic effect on bone	SC injection daily	Postmenopausal osteoporosis with high fracture risk	Increases lumbar BMD; reduces VCF in RCTs; increased fusion rate in animal models	Similar to teriparatide; limited ASD-specific data	Emerging alternative to teriparatide; further ASD surgery studies needed
Romosozumab	Dual (anabolic + antiresorptive)	Monoclonal Ab to sclerostin; activates Wnt pathway in bone formation + ↓ resorption	SC injection monthly × 12 months	Postmenopausal osteoporosis; high cardiovascular risk is contraindication	26% increase in lumbar HU after ~ 10.5 months; improved biomechanical parameters on FEA; increased fusion in animal models	Cardiovascular risk cost; limited ASD clinical data	Promising for pts needing rapid BMD gains; requires further clinical validation in ASD surgery

*BP* Bisphosphonate*, SERM* Selective estrogen receptor modulator, *RANKL* Receptor activator of nuclear factor kappa-B ligand, *PTH* Parathyroid hormone, *ZA* Zoledronic acid*, VCF* Vertebral compression fracture, *BMD* Bone mineral density*, FEA* Finite element analysis *PJK* Proximal junctional kyphosis, *SC* Subcutaneous, *IV* Intravenous*, ADT* Androgen-deprivation therapy *AI* Aromatase inhibitor, *ONJ* Osteonecrosis of the jaw

### Calcitonin

Calcitonin is a hormone which inhibits bone resorption via osteoclasts, thereby decreasing serum calcium. This medicine, derived from fish, is used for postmenopausal osteoporosis. Several animal studies have examined the relationship between fusion rates using this medicine and control or bisphosphonates and found mixed results [48, 49]. Clinical studies in spine fusion are lacking.

### Bisphosphonates

Bisphosphonates (BP’s) are antiresorptive medications that inhibit bone resorption by inhibiting osteoclasts, blocking osteoclastic binding sites on hydroxyapatite, and encouraging osteoclast apoptosis. They remain in bone for decades and lead to increased mineralization of the bony matrix, but do not increase bone volume. They are well-tolerated with few side effects, but patients’ fear of rare complications of atypical femoral fractures and osteonecrosis of the jaw have limited use [50].

Animal studies of BP’s in the setting of spine fusion are mixed with studies showing poorer outcomes with BP treatment, no effect, or increases in fusion mass, volume, density and strength [51]. Clinical studies indicate that BP’s do not inhibit fusion, and there is weak evidence that they may enhance outcomes. Nagahama et al. compared alendronate to vitamin D in a randomized controlled trial (RCT) of osteoporotic patients in the setting of single level posterior lumbar interbody fusion (PLIF) and found higher fusion rates, less cage subsidence, and fewer vertebral compression fractures (VCF) in the alendronate group [52]. A triple blinded RCT with zoledronic acid (ZA) compared to placebo found superiority in fusion rate and VCF after PLIF for ZA [53]. A retrospective review of 64 lumbar interbody fusion patients who received ZA or placebo found higher fusion rate, and lower incidence of VCF’s, cage subsidence and pedicle screw loosening in the ZA group [54]. Patient reported outcomes (PRO’s) in the ZA group also demonstrated greater improvement, perhaps due to fewer complications. Other studies have found no differences between BP treatment and placebo postoperatively [54, 55].

Bisphosphonates appear to be effective in reducing some mechanical complications, but many studies do not show a difference in outcomes when treated. Thus, it is recommended that BP’s be continued in the setting of adult spinal deformity surgery.

### Selective estrogen receptor modulators

Selective estrogen receptor modulators (SERM’s) inhibit osteoclast activation and bony resorption by activating estrogen receptors in bone. They are used to treat osteoporosis and have been demonstrated to decrease compression fractures and prevent bone loss in women [56]. Animal studies comparing raloxifene with BP’s or placebo have had variable results, with some indicating higher fusion rates in ovariectomized rats but not those with ovaries, and some without a difference [56, 57]. Clinical research is lacking. Future research is needed to understand the impact in human spinal fusion.

### Denosumab

Denosumab is a monoclonal antibody to RANKL that limits osteoclastic bone resorption. It is FDA approved for the treatment of osteoporosis and to prevent bone loss for patients receiving androgen-deprivation therapy or aromatase inhibitor therapy.

Tani et al. prospectively followed 21 patients with postmenopausal osteoporosis who were treated with denosumab postoperatively [58]. They assessed BMD over time and used finite element analysis (FEA) to model pedicle screw pullout strength increasing at 12 and 24 months of treatment postoperatively. Ide et al. examined denosumab in conjunction with teriparatide after PLIF. Both groups received teriparatide one month preoperatively and one year postoperatively; the denosumab group also received denosumab at months 2 and 8 postoperatively [59]. The fusion rate was higher in the combination group at 6 months than teriparatide alone (82% compared to 36%), but at the one year time point there was not a statistically significant difference (91% vs 64%). The combination group had higher BMD at one year and bone turnover markers BAP and P1NP were also found to be elevated. These results are relatively promising and warrant additional study.

### Romosozumab

Romosozumab is another monoclonal antibody that binds sclerostin. Sclerostin is an inhibitory factor in the Wnt signaling pathway; thus, binding it inhibits suppression of Wnt signaling [60]. The ensuing activation of Wnt leads to increased bone formation, decreased resorption, and increased bone mass and strength.

Animal studies has demonstrated romosozumab increased bone fusion rate and volume in rats, in osteoporotic and non-osteoporotic animals [61, 62].

Ishikawa et al. conducted a prospective study of osteoporotic patients undergoing 3 months of romosozumab treatment to characterize changes in the biomechanical parameters of their spine based on FEA; the control group was given eldecalcitol, a vitamin D analog [63]. The romosozumab group had greater increases in BMD and biomechanical parameters of compression strength, pull-out strength, and cage subsidence as measured by FEA. Mikula et al. opportunistically reviewed CT scans of patients treated with romosozumab and found at average of 26% increase in lumbar HU’s after an average of 10.5 months of treatment [64]. Further studies investigating the use of romosozumab in the setting of spine surgery are required.

### Parathyroid hormone receptor agonists

In humans, naturally occurring parathyroid hormone increases available serum calcium by increasing resorption of bone and renal reabsorption. Recombinant N-terminal parathyroid protein [teriparatide, rhPTH(1–34)] has an overall anabolic effect of stimulating osteoblasts greater than osteoclasts when administered intermittently. Teriparatide is used to treat osteoporosis and had demonstrated increased BMD, bone mass, bony mechanical strength in addition to reducing compression fractures [65, 66]. Animal studies have repeatedly showed earlier and better quality fusion with teriparatide treatment compared to control [51].

Teriparatide has been shown to reduced complications in deformity patients with osteoporosis. Mohanty et al. performed a retrospective review of patients undergoing > 7 instrumented levels spine surgery with two-year follow up [67]. Patients with osteoporosis were treated with teriparatide (20 µg/day) for at least 6 months preoperatively and one year postoperatively. They were compared to patients with osteopenia who received calcium and vitamin D and patients with normal BMD. Patients with osteoporosis treated with teriparatide had lower reoperation and pseudarthrosis rates compared to patients with osteopenia who were not treated at 2 years postop. In fact, the clinical and patient reported outcomes of the osteoporotic group treated with teriparatide were the same as patients with normal BMD.

**Fig. 1:**
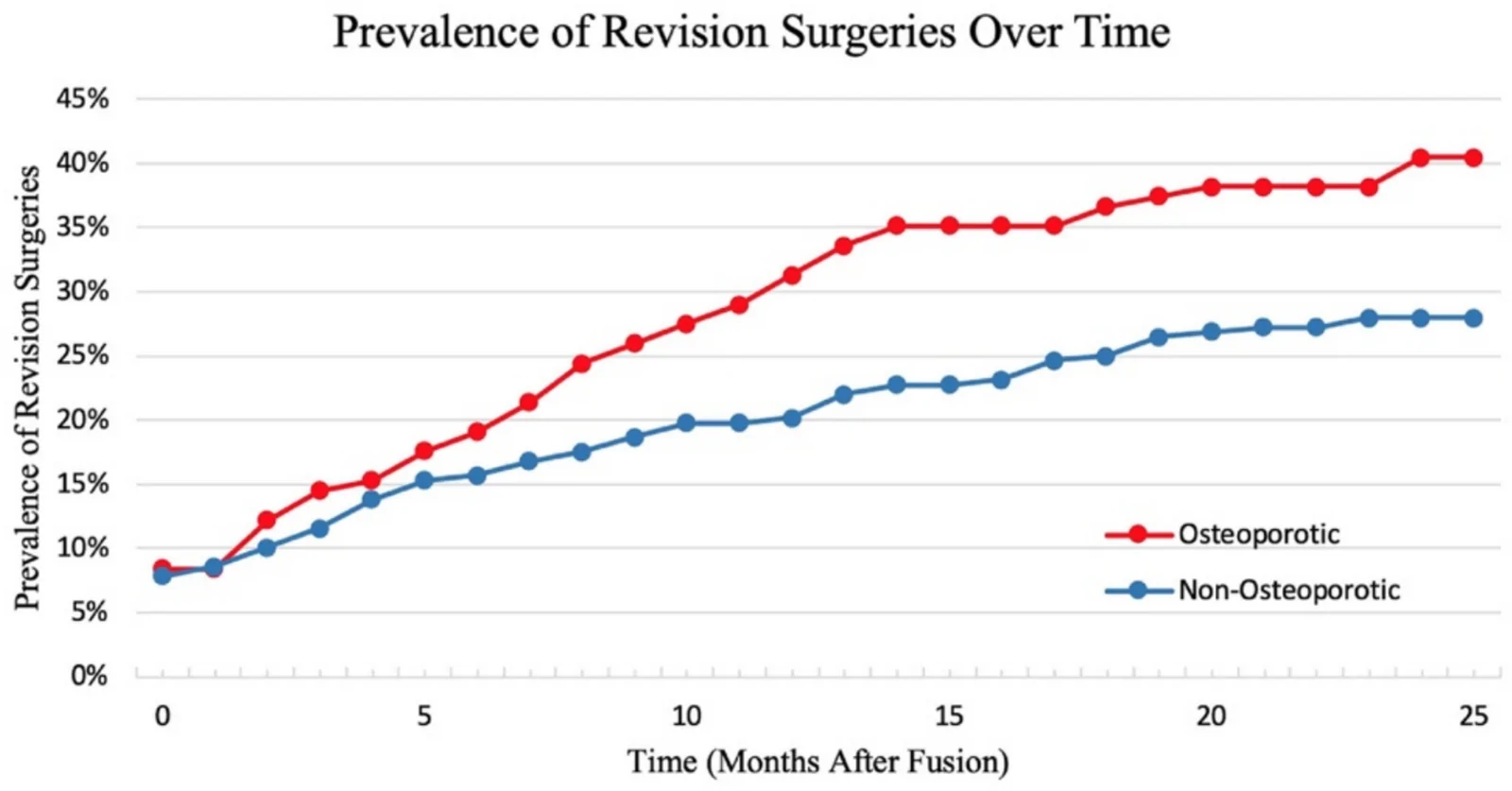
Prevalence of adult spinal deformity revision surgeries over time by osteoporosis status: reproduced from *Gupta *et al*. 2020* [40]

Ebata et al. demonstrated that teriparatide improves lumbar fusion rates in an RCT of 66 postmenopausal osteoporotic women who underwent PLIF or TLIF [68]. There were no differences in the PROs between the groups, although both improved significantly from baseline. Ohtori et al. reviewed 57 women who underwent PLIF in the setting of teriparatide or risendronate for 2 months preoperatively and 8 months postoperatively [69]. At 12 months the teriparatide group had greater fusion; their fusion rate was also faster at 8 months compared to 9.7 months as measured by CT. Again, there were no differences in PROs between the groups. Ushirozako et al. performed a retrospective review and found that there was significantly greater incidence of complete union in patients receiving teriparatide compared to control [70]. Another study by Ohtori et al. confirmed improved and earlier fusion with teriparatide compared to BPs, and also revealed that long duration of therapy improved these results further [71]. A contrasting study by Cho et al. compared PLIF patients on teriparatide and alendronate or alendronate alone. They did not fine a difference in fusion rates or PROs up to 24 months, but did observe earlier fusion in the teriparatide group (6.0 vs 7.2 months) [72]. They also assessed pedicle screw loosening (PSL) and cage subsidence and found no differences. Ohtori et al. conducted a prospective study looking specifically at PSL in the setting of PLF in osteoporotic women. The groups compared were teriparatide, risedronate or no medication for 2 months preoperatively and 10 months postoperatively. The teriparatide group and the least PSL (13%) which was significantly less than the risendronate group (26%) and control (25%) [73]. Yet again, there were no differences in PROs between the groups. Yagi et al. prospectively followed 43 patients who received teriparatide surgery after adult spinal deformity surgery and 33 patients who did not [74]. They found that BMD increased in the teriparatide group but decreased in the control. There was likewise an increase in volumetric BMD and bone mineral content at UIV + 1 in the teriparatide group. The treated group also had significantly decreased incidence of bony PJK compared to control at 2 years (4.6% vs. 15.2%) Fig. 1.

An expert consensus study suggests anabolic agents such as teriparatide for osteoporotic patients undergoing spine fusion [75]. Teriparatide has been shown to increase bone formation and fusion; the data above suggest that preoperative and postoperative use helps achieve faster and more reliable lumbar spine fusion compared to BP or placebo [76].

Abaloparatide is another parathyroid hormone analog, however with fewer studies performed to date, particularly in the operative osteoporotic ASD population. An animal study demonstrated an increased rate of bilateral posterior lateral fusion rate in rats at 28 days postop [77]. In postmenopausal women with osteoporosis, Matsumatoto et al. conducted an RCT and found an increase in lumbar spine BMD in the abaloparatide group [76]. Similarly, Miller et al. conducted an RCT in postmenopausal women with osteoporosis and found an increase in BMD along with fewer vertebral compression fractures in the treatment group [78].

### Surgical strategies

Data on surgical strategies for optimization of adult spinal deformity surgery in the specific setting of osteoporosis are limited. The primary risks of osteoporosis, as described previously, are fracture, failure of fusion, or failure of the bone screw interface. Various techniques such as supplemental fixation, modifying screw techniques have been suggested in the deformity setting, with few focusing on ASD [79].

### Bone cement augmentation

Screw loosening is a particular concern in osteoporotic bone. Pedicle screw augmentation with polymethylmethacrylate (PMMA) or other calcium or magnesium based bone cements at the upper and/or lower instrumented vertebra has been proposed. In this technique, the cement is either injected into the pedicle tract directly and then the screw inserted, or a fenestrated pedicle screw is used with the cement injected through the screw. In a non-deformity cadaveric setting, PMMA screws have demonstrated increased pullout strength compared to expansile screws in osteoporotic bone [80]. A recommended dose of 2-3 cc per level provides pull-out strength advantage while minimizing risk of extrusion [81]. Lee et al. examined 51 osteoporotic patients who had undergone three or more levels of lumbar fusion and found that the rate of radiographic adjacent segment degeneration was higher in those who had a cement-augmented UIV than those who did not at 2 years postop [82]. There was no difference in long-term PJF rates or revision surgery. Data on cement augmentation in ASD that is not exclusive to patients with osteoporosis can be considered as these studies often have patients with a various bone densities. Ghobrial et al. conducted a retrospective review of 85 patients, with and without osteoporosis, who underwent long segment fusion over five levels for adult spinal deformity [83]. Their two groups included patients who had PMMA at UIV and UIV + 1 compared to those who did not have cement augmentation. They found a lower rate of PJK, a lower UIV + 2 angle, and a lower rate of revision for PJF in the cement group. This is in spite of the cement group having lower T-score and more patients with osteoporosis/osteopenia. Theologis and Burch retrospectively reviewed 51 ASD patients who received cement augmentation at UIV and UIV + 1, no cement, or cement at variable levels at the proximal end of the construct [84]. The two levels of cement group had fewer revisions for PJF compared to the other cohorts. They also found improved PRO’s in the two level cement group. Risks of cement include leakage, risk of pulmonary embolism, and nidus for infection.

### Expandable screws

Another strategy to minimize screw loosening is use of pedicle screws with an expandable distal end designed to increase pullout strength [85]. Holler et al. conducted a study comparing 6.0 mm expandable screws to nonexpendable in cadaveric specimens and found a significantly greater failure load for the expandable screws [86]. Wu et al. reviewed 157 osteoporotic patients who underwent fusion for degenerative conditions; 80 with expandable screws and 77 with standard [87]. At two years, they found a rate of 4.1% loosening and 0.4% breakage in the expandable group. In the standard group, the loosening rate was 12.9% with no breakages. More data are needed on these screws.

### Tethers

Proximal junctional failure may be due to fracture, spondylolisthesis, implant failure or soft tissue failure, augmentation of posterior soft tissue envelope has been proposed as a method of decreasing its incidence. The stability of this tension band may minimize screw loosening and/or stabilize the adjacent segment. The interplay between the soft tissue tension band and osteoporosis requires further research, but studies of ASD patients with various bone densities can be considered. Rodriguez-Fontan et al. reviewed 80 ASB patients, 20 who had Mersilene tape augmentation to UIV and UIV + 1 and 60 with no augmentation [88]. At two years, they found a PJK rate of 15% in the augmented group and 38% in the unaugmented. In the subgroup of patients with osteoporosis, these rates were 14% in the augmented and 57% in the unaugmented. A review of tether literature by Sursal et al. found that 14 of 15 articles favored tethers to reduce PFK or PJF and one found no difference [89]. Notably, patients had various bone densities and various types and techniques of tethers were used.

### Recommended screening approach for ASD surgery

Given the high prevalence of osteoporosis and osteopenia in the ASD population and it's impact on surgical outcomes, this task force recommends a systemic preoperative bone health screening protocol for all adult spinal deformity surgery candidates. All patients aged 65 and above should undergo bone density analysis. Patients with risk factors for low bone density should undergo DXA regardless of age. Opportunistic assessment using existing preoperative CT scans (Hounsfield units) and MRI (VBQ) should be incorporated whenever these studies are available. Given that standard lumbar DXA is frequently unreliable in ASD patients due to deformity artifact, total hip and distal radius measurements should be prioritized. Complete spine imaging should be scrutinized for remote compression fractures which may also yield a clinical history of osteoporosis.

Spine surgeons are encouraged to establish collaborative pathways with metabolic bone disease specialists such as endocrinologists. While spine surgeons may not feel comfortable managing laboratory screening for ASD patients, endocrinologists typically order a panel of 25-hydroxyvitamin D, serum calcium, PTH, thyroid stimulating hormone, complete metabolic panel, complete blood count, and albumin/prealbumin for screening. This multidisciplinary approach is critical as endocrinologists often have workflows for arduous task of obtaining insurance authorization for osteoporosis medications.

When possible, a collaborative pathway consisting of bone density analysis, laboratory analysis, and preoperative medical optimization should be established.

## Conclusion

Adult spinal deformity in the setting of osteoporosis is challenging, but surgery offers patients significant quality of life improvements. Optimizing surgical outcomes and minimizing surgical risks is mandatory. New diagnostic tools to assess bone density, medical treatments to improve bone density and quality, and surgical strategies to minimize complications continue to evolve. Costs are considerable, both for medical and surgical treatment with coverage varying by insurance. There is no consensus on the gold standard of pre or intraoperative optimization for these patients. This task force recommends, at minimum, assessment of bone density, treatment with an anabolic agent to increase bone density for at least 3 months prior to ASD surgery in patients with osteoporosis, and preoperative planning to optimize alignment. This taskforce also recommends continued investigation into adjuvant medical and surgical strategies for this high-risk population.
